# Supplementary figures and images for: Global accumulation of circRNAs during aging in Caenorhabditis elegans
Source: BMC Genomics. 2018 Jan 3;19:8. doi: 10.1186/s12864-017-4386-y (PMC5753478; doi:10.1186/s12864-017-4386-y)

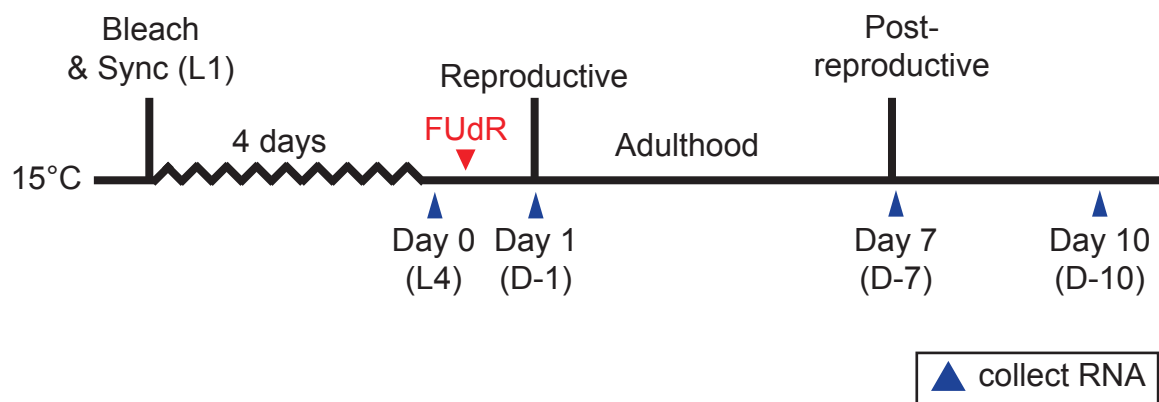

**FIG. S1**

Supplement: Supplementary file 1 — C. elegans aging paradigm. Protocol for collecting total RNA during C. elegans aging. Wild-type animals were fed E. coli OP50 and grown at 15°C. Gravid adults were bleached and populations were synchronized as L1 larvae and grown for an additional 4 days. At the L4 larval stage (Day 0), animals were either collected or transferred to FUdR containing NGM agar plates seeded with E. coli OP50, and were allowed to continue growth at 15°C. Total RNA was collected at different age time-points (L4, D-1, D-7, and D-10). (PDF 107 kb) [file 12864_2017_4386_MOESM1_ESM.pdf]

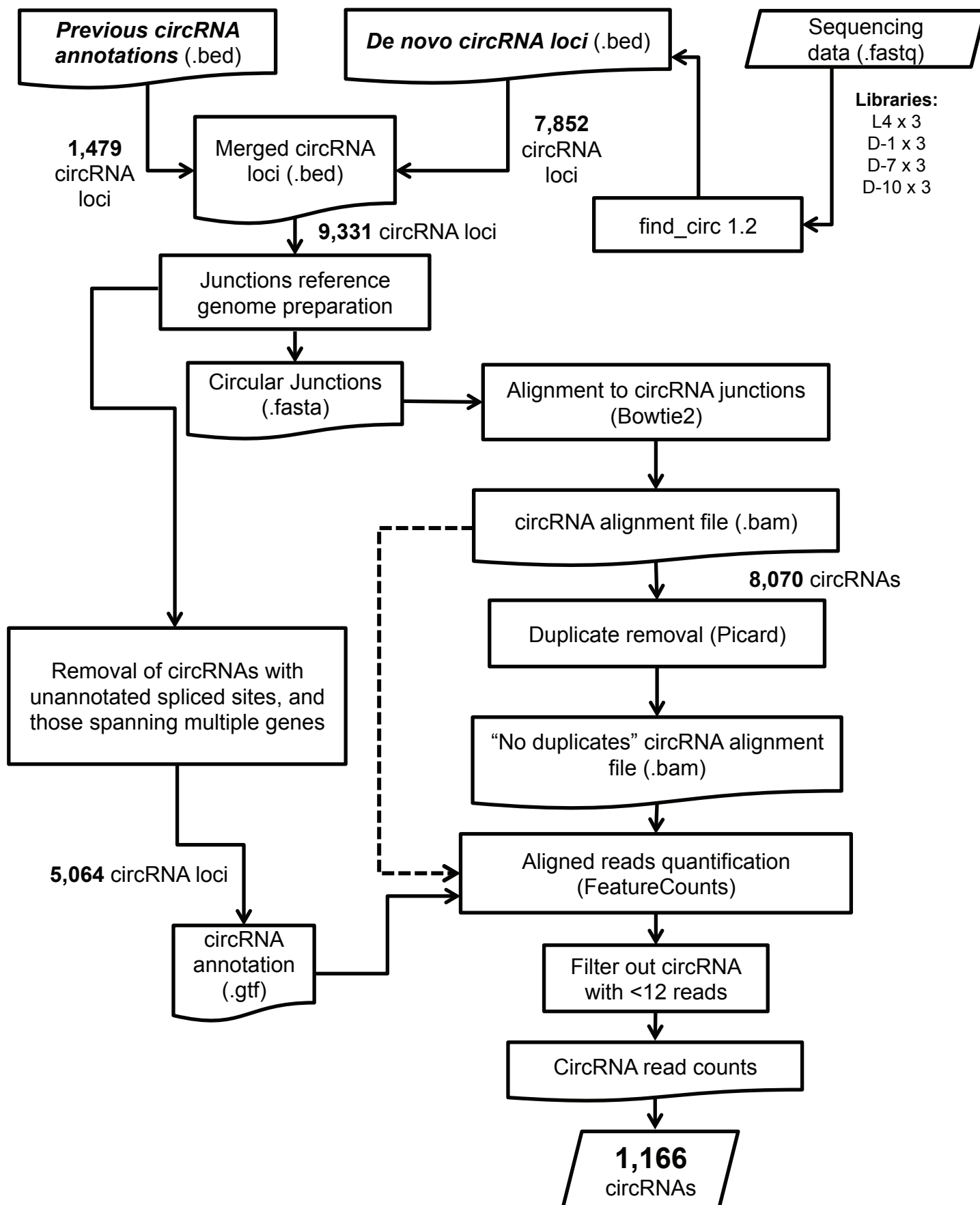

**FIG. S2**

Supplement: Supplementary file 3 — Pipeline for circRNA annotation. A flowchart of the computational pipeline used for circRNA identification. (PDF 138 kb) [file 12864_2017_4386_MOESM3_ESM.pdf]

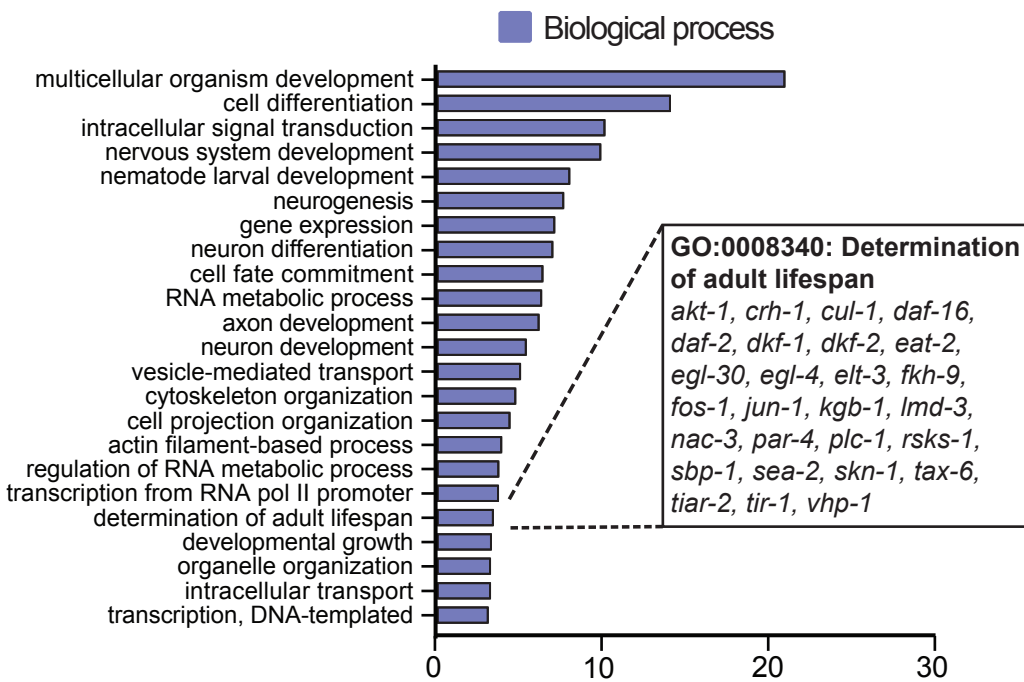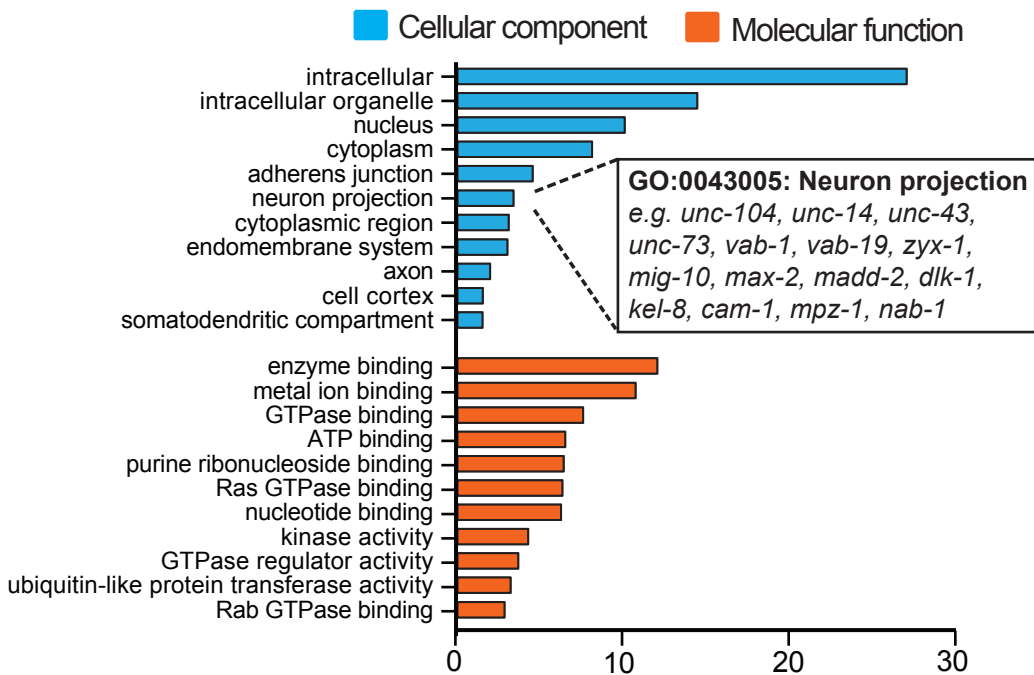

**FIG. S3**

Supplement: Supplementary file 8 — GO analysis of circRNA host genes. Visualization of ClueGO analysis of the 797 genes harboring the 1166 expressed circRNAs. X axis represents -log(P-value) of the enrichment. A Complete GO analysis is found in Additional file 9: Table S5. (PDF 141 kb) [file 12864_2017_4386_MOESM8_ESM.pdf]
